# Signaling pathways in Parkinson’s disease: molecular mechanisms and therapeutic interventions

**DOI:** 10.1038/s41392-023-01353-3

**Published:** 2023-02-21

**Authors:** Xu Dong-Chen, Chen Yong, Xu Yang, ShenTu Chen-Yu, Peng Li-Hua

**Affiliations:** 1 College of Pharmaceutical Sciences, Zhejiang University, 310058 Hangzhou, P. R. China; 2 State Key Laboratory of Quality Research in Chinese Medicine, Macau University of Science and Technology, Macau, P. R. China

**Keywords:** Blood-brain barrier, Diseases of the nervous system, Regeneration and repair in the nervous system, Neurodevelopmental disorders

## Abstract

Parkinson’s disease (PD) is the second most common neurodegenerative disease worldwide, and its treatment remains a big challenge. The pathogenesis of PD may be related to environmental and genetic factors, and exposure to toxins and gene mutations may be the beginning of brain lesions. The identified mechanisms of PD include α-synuclein aggregation, oxidative stress, ferroptosis, mitochondrial dysfunction, neuroinflammation, and gut dysbiosis. The interactions among these molecular mechanisms complicate the pathogenesis of PD and pose great challenges to drug development. At the same time, the diagnosis and detection of PD are also one of obstacles to the treatment of PD due to its long latency and complex mechanism. Most conventional therapeutic interventions for PD possess limited effects and have serious side effects, heightening the need to develop novel treatments for this disease. In this review, we systematically summarized the pathogenesis, especially the molecular mechanisms of PD, the classical research models, clinical diagnostic criteria, and the reported drug therapy strategies, as well as the newly reported drug candidates in clinical trials. We also shed light on the components derived from medicinal plants that are newly identified for their effects in PD treatment, with the expectation to provide the summary and outlook for developing the next generation of drugs and preparations for PD therapy.

## Introduction

**Fig. 1:**
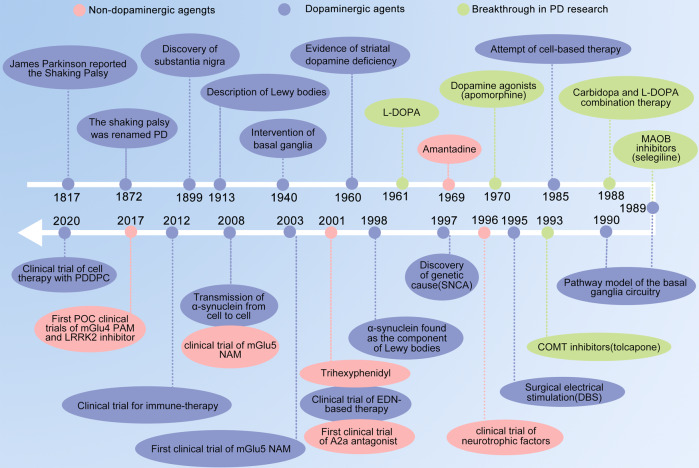
Basic research and drug development history for PD disease and therapy. A2a adenosine receptor type 2a, mGlu metabotropic glutamate receptor, NAM negative allosteric modulator, PAM positive allosteric modulator, EDN embryonic dopamine neuron, PDDPC personalized iPSC-derived dopamine progenitor cell, iPSC induced pluripotent stem cell, DBS deep brain stimulation

PD is the second most common neurodegenerative disease worldwide, with global prevalence increasing by 74.3% between 1990 and 2016.^1^ In 1817, James Parkinson published his monograph titled An Essay on the Shaking Palsy which represents the first description of PD as a neurological disorder.^2^ Beginning with Jean-Martin Charcot, a succession of scientists contributed to the comprehensive description of the clinical range and anatomopathological basis of PD, including motor, non-motor symptoms, the neuropathological changes in the substantia nigra (SN), Lewy bodies, and the role of dopamine (DA).^3,4^ Following these discoveries (Fig. 1), highly efficacious therapies like pharmacological DA substitution (levodopa treatment) and deep brain stimulation have become available to effectively control the symptoms. However, none of these treatments can stop PD from being a progressive disorder especially the increasing severity of treatment-resistant motor and non-motor symptoms still carry PD patient’s painful life.^5^ In this review, we described the clinical features and diagnostic criteria of PD, summarized the molecular mechanisms underlying PD and the research models, we also listed drugs used in market and clinical practice, and discussed available PD treatments like natural compounds, with the expectation to provide the summary and outlook for developing the next generation of drugs and treatments for PD therapy.

## Epidemiology, clinical features, and diagnostic criteria

The incidence of PD increases after 60 years of age, rising sharply to more than 3% among individuals of over 80 years old.^6–9^ In most populations, men have higher PD incidence than women.^8,10,11^ Variation in living habits and environment likely explains the difference of prevalence across regions and races. Environmental toxins may trigger PD symptoms, while dietary habits may alter disease incidence, notable examples include greater risk in smokers and people who regularly consume caffeine.^12–14^

Motor symptoms and non-motor symptoms make up the majority of the clinical characteristics of PD. Patients with PD have a variety of motor symptoms, including bradykinesia, muscle stiffness, rest tremor, and postural and gait difficulties.^15,16^ There are two main kinds of PD: tremor-dominant PD and non-tremor-dominant PD, based on clinical findings. In comparison to non-tremor-dominant PD, tremor-dominant PD is frequently linked to a slower pace of development and less functional impairment.^17^ Olfactory dysfunction, cognitive decline, constipation, depression, sleep problems such as excessive daytime drowsiness and rapid eye movement sleep behavior disorder, autonomic dysfunction, pain, and exhaustion are examples of non-motor characteristics. Non-motor symptoms frequently appear in the early stages of PD before the motor symptoms.^18,19^ PD progression will have certain problems, such as dyskinesia, psychosis, and motor and non-motor fluctuations, as well as a deterioration of the motor characteristics and long-term symptomatic therapy.^20^ According to reports, up to 80% of PD patients have freezing of the gait and falls after roughly 17 years of the disease, and up to 50% of patients say they have experienced choking. Dementia is also said to be more common in individuals who have had PD for at least 20 years.^20^ The primary pathogenic characteristics of PD include the steady degradation of just a subset of neurons within particular brain areas, such as the SN, as the illness progresses. Dopaminergic neurons are lost exclusively in the ventrolateral SN in the early stages; however, this damage spreads in the late stages.^21–24^ Furthermore, in several different areas of the brain, certain neurons have abnormally high levels of α-synuclein accumulated in their cytoplasm.^25^ In cholinergic and monoaminergic brainstem neurons as well as olfactory neurons, aggregated α-synuclein produces Lewy bodies, a frequent hallmark of neuropathology. Lewy bodies multiply as Parkinson’s disease advances, impacting non-dopaminergic neurons in other brain regions in addition to the limbic and neocortical regions.^26,27^ Finally, neurons outside of the central nervous system (CNS), such those in the olfactory bulb or mesenteric system, deteriorate as a result of PD. Overall, 10% of identified patients were initially categorized as other illnesses due to the possibility that PD symptoms might manifest early and the complexity of the disease presentation.^28^ In order to increase diagnostic precision, the International Parkinson and Movement Disorder Society has established criteria. According to these recommendations, the presence of bradykinesia and at least one other cardinal motor characteristic constitutes Parkinson’s syndrome (4–6-Hz rest tremor or limb rigidity). Additionally included were “red flags” for alternate diagnosis and excluding clinical characteristics. DA transporter-single-photon emission computed tomography (DAT-SPECT), structural magnetic resonance imaging (MRI), magnetic resonance diffusion-weighted imaging (MR-DWI), and genetic testing are frequently utilized to make a clinical diagnosis of PD. Because hyposmia or anosmia are present in around 90% of individuals with PD, olfactory function testing with the UPSIT or Sniffin Stick tests is occasionally a component of the initial clinical examination.^29–31^ With the use of cutting-edge MRI methods, certain MRI characteristics that are highly specific for atypical parkinsonism have been identified. These include quantitative susceptibility mapping (QSM), which allows for the determination of iron accumulation in the SN, and neuromelanin imaging (NMI), which takes use of the paramagnetic characteristics of neuromelanin.^32,33^ Notably, NMI has the potential to show alterations in prodromal PD.^34^ A further technique used to distinguish between PD and clinical mimics unrelated to presynaptic nigrostriatal terminal dysfunction is ^123^I-ioflupane single-photon emission CT (SPECT).^35,36^ Despite these developments, one area of clinical diagnosis still needs work: the use of genetic testing, which is presently reserved for situations in which a particular hereditary etiology is suspected. However, we also notice a rise in the number of genes linked to complex symptoms that include parkinsonism as PD worsens. For such circumstances, routine genetic testing may be helpful.

## Etiology and pathogenesis of PD

### Environment genetic factors

The complex etiology of PD involves both environmental and genetic factors.^37,38^ Environmental causes are situations such as pesticide exposure, physical inactivity, head injury, and stress.^39^ People intoxicated with 1-methyl-4-phenyl-1,2,3,6-tetrahydrodropyridine (MPTP) developed a syndrome nearly identical to PD and its active metabolite, 1-methyl-4-phenylpyridinium (MPP^+^), is similar to paraquat in structure.^40^ Human epidemiological studies have implicated residence in a rural environment and related exposure to herbicides and pesticides with an elevated risk of PD.^41^ However, it is still necessary to get convincing data to implicate the link between specific toxins and PD.

### Autosomal-dominant PD genes

It has been demonstrated in patients with SNCA mutations whose brains showed the aggregation of α-synuclein, represented as the occurrence of Lewy bodies and the loss of DA neurons, that the PARK1/PARK4 gene for the expression of α-synuclein is related to the abnormal pathological aggregation of insoluble α-synuclein fibril.^42,43^ The most frequent genetic cause of PD is a mutation in the leucine-rich repeat kinase 2 gene (LRRK2), known as PARK8. LRRK2 mutations have primarily been found in late-onset individuals older than 50 years.^44,45^ The most common variations of this mutation are G2019S, R1441C, R1441G, and R1441H, which can cause DA neurons to die and degenerate by interfering with a variety of physiological processes, including vesicle transport, cytoskeletal function, protein synthesis, and the lysosomal system.^45,46^ It has been discovered that PARK13, the HTRA2 serine peptidase 2 gene (HTRA2), is released into the cytoplasm from damaged mitochondria and is crucial to maintaining normal mitochondrial function. It has also been demonstrated to play a neuroprotective role under stressful conditions, with PARK13 knockout mice exhibiting elevated levels of reactive oxygen species (ROS), mitochondrial dysfunction, and PD phenotypes.^47–49^ Its activity is controlled by PINK1-mediated phosphorylation, which is crucial for maintaining mitochondrial integrity under stress. PARK13 targets the destruction of misfolded SNCA.^50,51^ PARK17, a gene that makes up the reverse transcriptome complex (VPS35), is required for the retrotransfer of proteins from endosomes in the pre-lysosomal compartment network to the trans-Golgi network.^52^ The cation-independent mannose 6-phosphate receptor (CI-MPR) may bind with VPS35 in an endosomal compartment and get sequestered in recycling tubules, preventing it from being sent to vacuoles or lysosomes.^53^ In response to diverse conditions, the PARK18 gene, which encodes the eukaryotic translation initiation factor 4 gamma 1 (EIF4G1), controls the commencement of the translation of mRNAs encoding mitochondrial, cell survival, and growth proteins.^54,55^ The eIF4G1-eIF4E or eIF4G1-eIF3e binding, which is assumed to serve as the molecular bridge between the mRNA cap-binding complex and the 40S subunit and causes mitochondria-related imbalance, has been discovered to be affected by two mutations, EIF4G1 p.A502V, and EIF4G1 p.R1205H.^54,56^

### Autosomal-recessive PD genes

The most frequent cause of autosomal-recessive early-onset Parkinson’s syndrome is PARK2, which is encoded by the parkin RBR E3 ubiquitin protein ligase gene (PRKN). According to studies, the PARK2 mutation is present in up to 7% of PD patients between 30 and 35 years old and as much as 50% of PD cases over 25 years old.^57,58^ In the ubiquitin–proteasome system, which is thought to be a multipurpose neuroprotective agent against a variety of toxic injuries, including mitochondrial poisons, and is thought to be essential for the survival of DA neurons, Parkin plays a significant role as an E3 ubiquitin ligase, working in conjunction with E1 ubiquitin-activating enzyme and E2 ubiquitin-conjugating enzyme to degrade targeted proteins.^59^ It has been demonstrated that PARK6, a serine/threonine protein kinase encoded by the PTEN-induced putative kinase 1 gene (PINK1), interacts with parkin to encourage selective autophagy in depolarized mitochondria and maintain mitochondrial integrity.^60^ Parkin is typically recruited to depolarized mitochondria to start autophagy and eliminate the damaged or malfunctioning mitochondria after being phosphorylated by PINK1 to activate its E3 ligase activity.^37^ In inefficient mitochondria, PINK1 builds up in the outer mitochondrial membrane to start the removal of damaged mitochondria from the cell because it cannot be transmitted to the inner mitochondrial membrane to be broken down.^61^ PARK7, also known as DJ-1, the parkinsonism-associated deglycase gene, guards DA neurons in the model system from harm brought on by mutant synuclein, rotenone, 6-hydroxydopamine (6-OHDA), and hydrogen peroxide.^62^ The primary function of the widely expressed protein DJ-1 is to protect cells against oxidative stress through a variety of ways.^61,63^ According to reports, PARK2, PARK6, and PARK7 are all involved in the same biological process. Transmembrane endo-/lysosomal related proteins are encoded by PARK9, the ATPase 13A2 gene (ATP13A2). The lysosomal signaling lipids phosphatidylic acid and phosphatidylinositol (3,5) biphosphate interact with the N-terminus of ATP13A2, which serves as the catalyst for ATP13A2 action and controls endo/lysosomal cargo sorting.^64,65^ The majority of ATP13A2 mutations affect its functional domains, particularly its transmembrane and E1-E2 adenosine triphosphatase domains. If ATP13A2 is functionally lost, this may lead to Zn^2+^ dysregulation and abnormal cell metabolism, including dysfunctional energy production and decreased lysosomal proteolysis.^66–68^ In addition, it has been demonstrated that ATP13A2 reduces the neurotoxicity of α-synuclein.^69^ The PARK15 protein, encoded by the F-box protein 7 gene (FBXO7), is a subunit of the F-box protein that functions as an adapter protein in the SKP1/cullin-1/F-box protein E3 ubiquitin ligase complex to recognize and mediate the non-degrading ubiquitination of glycogen synthase kinase (GSK)-3β and the translocase of outer mitochondrial membrane 20 to control mitophagy, mitochondria motility, mitochondria membrane potential, mitochondria bioenergetics, mitochondria biogenesis and mitochondria-associated apoptosis.^70–74^ In addition, because FBXO7 is a stress-responsive protein, malfunction may lower complex-I’s activity in the electron transport chain, lowering mitochondrial membrane potential and ATP levels while raising cytoplasmic ROS.^75^

## Molecular mechanisms of PD

### α-synuclein aggregation

Neuronal degeneration has been linked to numerous molecular and cellular changes, including α-synuclein aggregation, aberrant protein handling, excitotoxicity, oxidative stress, apoptosis, and mitochondrial dysfunction. Abnormal α-synuclein aggregation is one of the most important hypotheses explaining the death of nigrostriatal neurons in PD.^76^ Localized to the cytosol, mitochondria, and nucleus, α-synuclein is a potential chaperone that plays a role in the dynamics of synaptic vesicles, intracellular trafficking, and mitochondrial function.^77–79^ Some evidence suggests that the protein participates in lipid metabolism of the brain, a process that contributes to PD pathogenesis.^80^ α-synuclein itself can become neurotoxic when soluble α-synuclein monomers form oligomers, which combine into tiny protofibrils and eventually form large, insoluble fibrils.^81,82^ Age-related decline in proteolytic defense mechanisms of the brain may play an important role in α-synuclein accumulation.^83,84^ Specifically, intracellular α-synuclein homeostasis is maintained by the ubiquitin–proteasome and lysosomal autophagy systems. Extracellular proteases not part of either system are also implicated cleaving α-synuclein. Thus, impairment of these degradation systems may contribute to α-synuclein accumulation.

### Oxidative stress

**Fig. 2:**
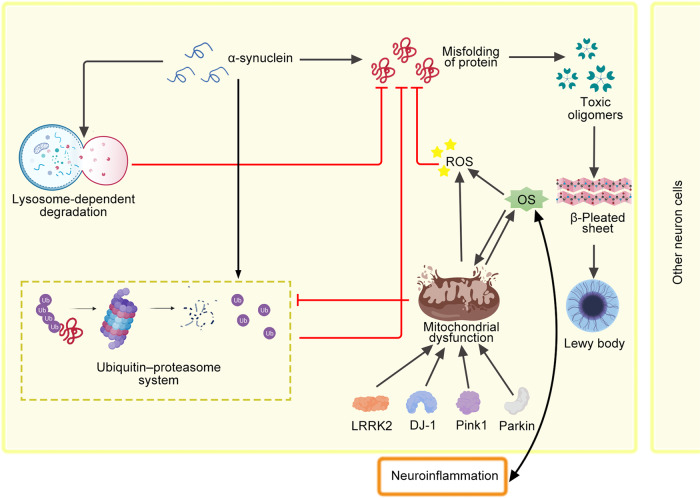
Intracellular α-synuclein homeostasis is maintained via the ubiquitin–proteasome and lysosomal autophagy systems. Impairment of these degradation systems by OS, mitochondrial dysfunction, or neuroinflammation could contribute to α-synuclein accumulation. Furthermore, mutations of genes like LRRK2, DJ-1, Parkin, and Pink1 cause mitochondrial dysfunction and increase cell death. Finally, OS and neuroinflammation appear to be connected

Oxidative stress (OS) is a major process in aging that directly harms the CNS. Under physiological conditions, free radicals or ROS are important to host defense, gene transcription, synaptic plasticity regulation, and apoptosis.^85^ However, OS occurs when ROS overwhelms cellular antioxidant activity. Cytotoxic compounds then accumulate to cause protein collapse, enzyme failure, lipid breakdown,^86^ and cell death in various neurons, including DA-neuronal tissue (Fig. 2).^87^ These dysfunctions contribute to PD pathogenesis, and may also be a cause of Alzheimer’s disease (AD).^88,89^ Currently, NADPH oxidase (NOX) is considered the most important ROS generator,^90^ playing a crucial role in triggering OS and neurotoxicity.^91^ Mitochondria are also major producers of ROS.^92,93^ The electron transport chain’s complexes I and III are thought to be where most ROS is produced in mitochondria. Superoxide radical, the main ROS generated in mitochondria, was created when the one electron was transported from oxygen to oxygen. Superoxide dismutase 2 or MnSOD may convert the superoxide radical to hydrogen peroxide, which the catalase enzyme can subsequently detoxify. However, the Fenton reaction, which severely oxidizes DNA or lipids, can cause hydrogen peroxide to transform into a highly reactive hydroxyl radical in the presence of metal ions like Fe^2+^.^93,94^ The mechanism of ferroptosis is connected to the imbalance of iron ion homeostasis, implying a connection between ferroptosis and OS.^95^ Lipids may be oxidized by the Fenton reaction’s hydroxyl radicals to produce lipid peroxides, which can cause ferroptotic cell death.^96,97^ Depletion of glutathione, which worsens intracellular OS by promoting the buildup of lipid peroxides to trigger ferroptosis, is another biochemical sign of ferroptosis. In addition, increased OS can decrease lysosomes and harm the lysosomal autophagy system, connecting OS to the buildup of α-synuclein. Another hypothesis contends that extra cytosolic DA can simply be oxidized to create DA-quinones. Then, the DA quinine-modified α-synuclein may partially inhibit chaperone-mediated autophagy, causing α-synuclein to self-assemble.^98,99^ Meanwhile, the aggregate formation of intracellular α-synuclein increased mitochondrial OS.^100^

### Ferroptosis

**Fig. 3:**
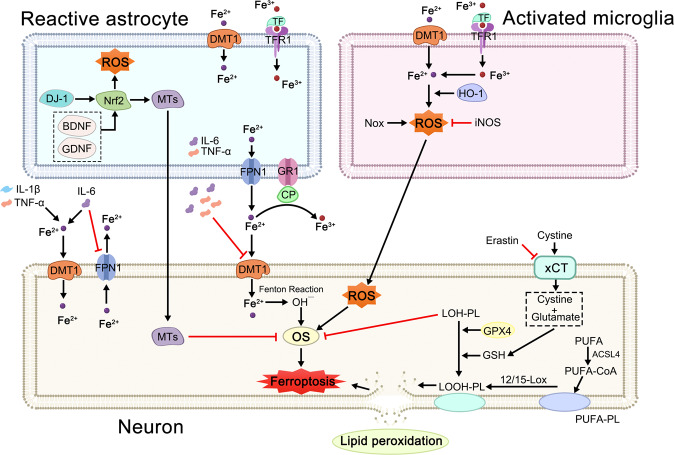
First, inflammatory cytokines (IL-1β, TNF-α, IL-6) released by activated microglia and astrocytes promote iron accumulation in neurons by upregulating DMT1 and downregulating FPN1. BDNF and GDNF secreted by activated astrocytes reduce iron accumulation in neurons by downregulating DMT1. Second, ROS released from activated microglia promote neuronal OS. Upregulation of Nrf2 and the release of metallothioneins in astrocytes contribute to neuronal resistance to OS. BDNF brain-derived neurotrophic factor, GDNF glial cell line-derived neurotrophic factor, HO-1 heme oxygenase-1, IL-1β interleukin-1β, IL-6 interleukin 6, iNOS inducible nitric oxide synthase, NOX NADPH oxidase, Nrf2 nuclear factor-erythroid factor-2, Tf transferrin, TNF-α tumor necrosis factor α, 12/15-LOX lipoxygenases 12/15, LOOH-PL lipid hydroperoxide–phospholipid, LOH-PL lipid alcohol–phospholipid

An aberrant iron metabolism and severe lipid peroxidation trigger ferroptosis, an iron-dependent kind of controlled cell death that results in OS and cell death (Fig. 3).^101^ It was also found to be involved in DA neuron death in PD.^102^ The enzyme acyl-CoA synthetase long-chain family member 4 (ACSL4) converts coenzyme A (CoA) to free polyunsaturated fatty acids (PUFA) in the cytosol. PUFA-CoA can then be incorporated into phospholipids, which are then oxidized by lipoxygenases 12/15 and cause lipid peroxidation. By halting lipid peroxidation, glutathione (GSH), an antioxidant that the body produces from glutamate and cysteine, can suppress ferroptosis.^103,104^ The rate-limiting substrate, cysteine, can either be produced from methionine via the transsulfuration route or taken up by the xCT antiporter as an oxidized cystine dimer. The xCT antiporter is inhibited by erastin, depleting the intracellular cysteine pool and impairing GSH production as a result. Another cellular antioxidant enzyme called DJ-1 prevents the transsulfuration pathway from being destroyed, protecting the production of cysteine and GSH and acting as a ferroptosis inhibitor.^105^ The only member of the glutathione peroxidase family capable of reducing lipid hydroperoxides under physiological circumstances is glutathione peroxidase 4 (GPX4).^106,107^ To convert lipid hydroperoxides to lipid alcohols, GPX4 requires decreased GSH, and one of the most popular methods to induce ferroptosis experimentally is the direct inactivation of GPX4 by RAS-selective lethal 3. The traits of ferroptosis induction are remarkably compatible with the pathogenic alterations seen in PD patients, and ferroptosis genes themselves can be connected to PD. Coexistence of iron and α-synuclein in Lewy bodies in the midbrain in PD patients.^108^ α-synuclein as a metal-binding protein will change the conformation while binding iron leading to the aggregation of α-synuclein.^109^ Iron homeostasis in neural networks is regulated by microglia and astrocytes. As a result of iron accumulation in activated microglia and subsequent production of proinflammatory cytokines, iron deposition in the CNS may rise. Divalent metal transporter 1 (DMT1), iron regulatory protein 1, and transferrin receptor 1 (TfR1) considerably elevated expression, but ferroportin 1 (FPN1) dramatically downregulated expression, which aggravated neuronal iron deposition.^110–115^ In addition to producing ROS, NOX in active microglia led to OS that caused DA neurons to undergo ferroptosis.^116^ Furthermore, inducible nitric oxide synthase (iNOS) was markedly elevated in microglia in response to inflammatory signals, which allowed it to inhibit 15-lipoxygenase activity and thwart ferroptosis.^117^ Various forms of iron are transported by astrocytes mostly through protein interaction cascades, particularly ceruloplasmin (CP). Although CP’s ferroxidase activity may efficiently oxidize Fe^2+^ into Fe^3+^, enabling iron efflux from cells, almost 80% of this activity was reduced in the SN of PD patients, suggesting that decreased CP expression and consequent iron buildup play a role in neuronal mortality in PD.^118–121^ Iron buildup in the brain that is neurotoxic will also be aided by microglia and astrocytes overexpressing heme oxygenase-1.^122,123^ To remove ROS from DA neurons, reactive astrocytes may also produce different antioxidant molecules like GSH and metallothioneins. Nrf2 activation in astrocytes can also upregulate antioxidant enzymes including GSH production enzymes and MTs to protect the DA neurons from OS.^124^

### Mitochondrial dysfunction

Mitochondrial dysfunction is increasingly understood to be important in PD pathogenesis. Indeed, many studies have found that mitochondrial dysfunction induces dopaminergic neurodegeneration and chronic ROS production. The first indication of this connection arose from observations after infusions of MPTP selectively inhibited mitochondrial complex I.^40,125^ The same negative outcomes occurred with other inhibitors of this complex, including rotenone, pyridaben, fenpyroximate, and trichloroethylene.^126–128^ In addition, mice overexpressing α-synuclein were more susceptible to toxins than α-synuclein-knockout mice, suggesting that mitochondrial α-synuclein worsens toxicity.^129–131^ Transcription-factor dysregulation and the resultant changes to mitochondrial biogenesis are hypothesized to be major causes of mitochondrial dysfunction. In particular, transcription-factor coactivator peroxisome proliferator-activated receptor gamma coactivator-1a (PGC-1a) is a key regulator of mitochondrial biogenesis. In PGC-1a knockout mice, dopaminergic cells are more sensitive to MPTP, whereas PGC-1a overexpression protects against neurotoxicity.^132,133^ Another source of mitochondrial dysfunction is pathogenic mutations in certain genes, such as Parkin, DJ-1, LRRK2, and PINK1.^126,131,134,135^ Parkin encodes E3, a ubiquitin protease ligase. Animals lacking Parkin are highly susceptible to rotenone, a mitochondrial complex-I inhibitor.^131,135,136^ Mutations to PINK1 cause an autosomal-recessive form of PD, likely through decreasing mitochondria respiration and ATP synthesis, while increasing α-synuclein aggregation.^137^ PINK1 dysfunction also appears to cause defects in mitochondria localization and impairs mitophagy.^138^ Research on combined Parkin and PINK1 knockouts in *Drosophila* showed that they belong in the same pathway, with PINK1 being upstream to Parkin.^59^ When mitochondria are damaged and depolarized, the cytosol recruits Parkin to mediate selective autophagic removal.^139^ For Parkin translocation, dysfunctional mitochondria must accumulate PINK1 and activate kinases.^140–143^ Research suggests that Src homology 2 domain-containing tyrosine phosphatase-2 (SHP2) is important to mitochondrial translocation and ubiquitination of Parkin, given that SHP2 knockdown inhibits the process (Sun et al. ^144^). Tyr dephosphorylation may be the mechanism underlying SHP2 regulation of Parkin activity. The drug lovastatin enhances SHP2 activity and thus is a candidate for PD treatment. Loss-of-function mutations in the DJ-1 locus also cause a rare autosomal-recessive form of PD and increase susceptibility to OS-induced cell death. Both DJ-1 knockout mice and humans carrying DJ-1 mutations have mitochondria with impaired respiration.^145–147^ In contrast, autosomal-dominant PD is associated with mutations in LRRK2. The striatum of older homozygous LRRK2G2019S knock-in mice exhibited mitochondrial abnormalities, as did the DA neurons of *Caenorhabditis elegans* harboring G2019SLRRK2 mutations.^148,149^ In general, mitochondrial fission is associated with LRRK2 mutations mediated by dynamin-like proteins.^150^

### Neuroinflammation

Cellular and molecular investigations of postmortem human brains revealed neuroinflammation-related damage in patients with PD.^151–154^ Both innate and adaptive immune responses are involved in PD progression.^155–158^ As brain-resident innate immune cells, activated microglia upregulate nuclear factor kappa-B (NF-*κ*B) and NLR family pyrin domain-containing 3 (NLRP3), triggering an increase of cytokines, such as IL-1β and TNF-α.^159,160^ In patients with early PD, the midbrain and putamen are more densely populated with activated microglia,^161,162^ correlating with decreased activity of DA transporter ligands. Despite the widespread acceptance of chronic inflammation in PD, we remain uncertain regarding how neuroinflammation occurs. As a damage-associated molecular pattern (DAMP), α-synuclein may cause a proinflammatory shift when entering cells via toll-like receptor (TLR)-2.^159,163–166^ Dying or damaged cells may also release DAMPs, IL-1α, or mitochondrial ROS that trigger an innate immune response upon interaction with pattern recognition receptors (PRRs). Consecutive NLRP3 activation then elevates IL-1β synthesis, initiating further innate immune responses.^167^ Therefore, microglial activation in PD^168,169^ may result from PRR-mediated responses to DAMPs. In animal models with 6-OHDA-induced neurodegeneration, microglia were gradually repolarized from an anti-inflammatory M2 to a proinflammatory M1 phenotype.^170^ After repolarization, NF-*κ*B initiates cytokine production in M1 cells,^159,171,172^ leading to interleukin and procaspase-1 transcription. These processes form the inflammasome NLRP3, which works with caspase-1 to activate proinflammatory IL-1β. Other proinflammatory proteins released from M1 cells (e.g., iNOS and TNF) also contribute to neurodegeneration in PD.^173^ Finally, Noelker et al. observed that TLR-4 knockout lowered the number of activated microglial cells and protected against SN dopaminergic degeneration, demonstrating that TLR-4 contributes to neuroinflammation.^174^ Adaptive immune responses also factor into neuroinflammation during PD. Several studies indicate that T-cell subpopulations contribute to PD pathophysiology.^175^ For example, CD4 and CD8 T cells significantly infiltrated into the SN of patients with PD,160 with CD8 T-cell concentrations particularly high.^176,177^ Because this infiltration occurs in early-stage PD and subsides with disease progression, CD8 T cells seem to be important at the beginning of the disease. Further evidence of CD4 T-cell contribution to neurodegeneration include their population and activity shifts in patients with PD, along with an increase in human leukocyte antigen-DR positive antigen-presenting microglia.^178^ Studies on the role of Th17 cells in PD have confirmed this hypothesis. Neurons appear to be more susceptible to IL-17 or autologous Th17 cells and are eventually subjected to NF-*κ*B-dependent cell death.^179^ In addition, knockout or pharmacological inhibition of CD4 T cells downregulates major histocompatibility complex (MHC) II expression in CNS myeloid cells and protects against tyrosine hydroxylase (TH) neuron loss in the ipsilateral SN pars compacta (SNpc).^179^

### Gut dysbiosis

**Fig. 4:**
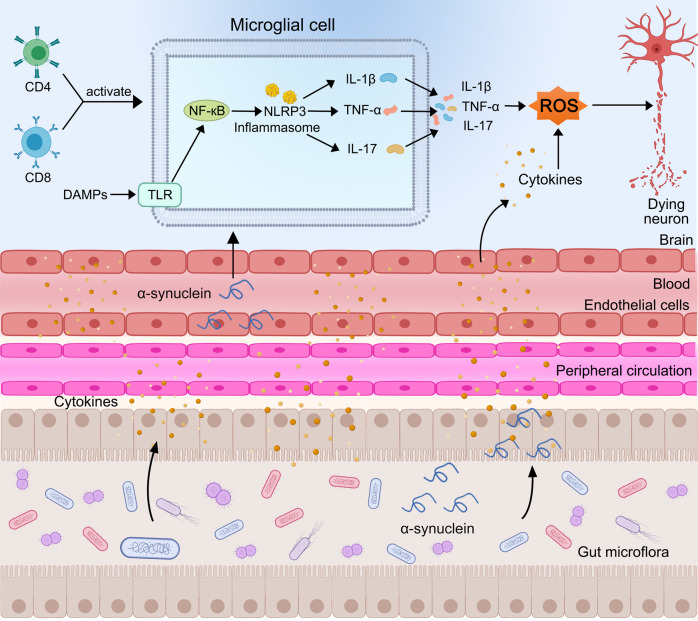
DAMPs (e.g., α-synuclein) trigger an innate immune response upon interaction with pattern recognition receptors in microglial cells. Microglial activation then increases the amount of NF-κB and NLRP3, leading to subsequent cytokine upregulation. Gut dysbiosis sends signals to the CNS and enteric nervous system via metabolites, hormones, and the immune system, thus mediating neuroinflammation

The role of gut microbiota in neurological diseases has attracted considerable interest. Gut–brain microbiota signaling encompasses the CNS, enteric nervous system, autonomic nervous system, and hypothalamic–pituitary–adrenal axis. Signaling pathways between the CNS and enteric nervous system involve metabolites, hormones, the immune system, and afferent nerves.^180,181^ Microbiota can mediate inflammation of the enteric nervous system (Fig. 4). Intestinal inflammation is a contributing factor in PD pathogenesis, as patients exhibit high levels of zonulin and alpha-1-antitrypsin, markers of intestinal barrier dysfunction, as well as of calprotectin, a marker of intestinal inflammation.^182^ Specific microbial taxa have been closely associated with systemic inflammatory responses. For instance, Verrucomicrobiaceae abundance was correlated with plasma interferon (IFN)-γ levels, while Bacteroides abundance was associated with plasma TNF levels. Roseburia upregulated innate immune genes and negatively regulated the NF-*κ*B pathway, thus promoting immune homeostasis.^183^ Through these effects on intestinal inflammation, gut microbiota and their metabolites could influence PD pathophysiology.^184^ A notable metabolite in this regard is lipopolysaccharide (LPS), which increases both α-synuclein accumulation in the enteric nervous system and intestinal permeability.^185^ Moreover, LPS intervention in Thy1-α-synuclein mice significantly decreased two tight junction proteins (zona occludens 1 and E-cadherin) in intestinal epithelial cells, highlighting a relationship between gut microbiota and PD pathogenesis.^186^ An example of evidence supporting microbiota involvement in PD is the finding that exposure to curli-producing bacteria increases α-synuclein deposition and accumulation in intestinal ganglion cells and the brain, leading to inflammation.^187^ Multiple animal experiments have also demonstrated that α-synuclein pathology can spread from the intestine to the brain along the gut–brain axis, and injecting α-synuclein into the intestinal wall causes pathological changes in the CNS.^188–191^

However, pathological processes are not necessarily limited to proceeding along the brain–gut or gut–brain axes. Pathologies may develop separately in the enteric nervous system and CNS during disease progression. In consideration of this possibility, Arotcarena et al. proposed a mechanism involving the general circulation acting as a path for long-distance, bidirectional transmission of endogenous α-synuclein between the intestinal tract and CNS.^192^

## Research models of PD

Our understanding of the pathophysiology, etiology, and molecular processes of PD has improved thanks to a variety of models.^193^ The SNpc dopaminergic neurons constitute the foundation of the SNpc, and toxins like 6-OHDA, MPP^+^, and MPTP may quickly degenerate the SNpc, resulting in strong, well-defined motor impairments. Toxin models are the traditional models for PD research.^194–196^ Animal models of PD span invertebrate and vertebrate animals. Although *C. elegans* overexpressing α-synuclein damages dopaminergic neurons, the degeneration is not progressive, and α-synuclein inclusions are lacking.^197,198^ *Drosophila* overexpressing wild-type (WT), A53T, and A30P α-synuclein showed many PD characteristics, including age-dependent, selective loss of dopaminergic neurons and Lewy body-like filamentous inclusions.^199^ Nevertheless, they do not express α-synuclein with the complexity of vertebrates, nor can these models exhibit key clinical features, such as resting tremors, bradykinesia, and rigidity. While transgenic PD mice do not exhibit overt degenerative pathology in dopaminergic neurons, functional abnormalities are present in their nigrostriatal system.^200^ Of the mouse models available, only the mouse prion promoter A53T α-synuclein transgenic mice (MitoPark) recapitulated the full range of α-synuclein pathology observed in humans.^200–203^ These MitoPark mice are thus particularly promising as PD models. They are generated through selective disruption of the gene encoding mitochondrial transcription-factor A (Tfam) in dopaminergic neurons. The mutation lowers mitochondrial DNA copy number, similar to characteristics observed in human PD.^204,205^ In addition, Tfam disruption causes a respiratory chain deficiency that results in a progressive degenerative phenotype. Both these features of mitochondrial dysfunction are present in human PD, again emphasizing the model’s usefulness. Recently, a novel model emphasizing the aggregated, misfolded forms of α-synuclein seen in Lewy bodies has emerged as a crucial tool in PD research. Preformed fibrils (PFFs), which resemble the structural components of Lewy bodies and Lewy neurites, were produced by researchers incubating recombinant α-synuclein monomeric proteins under certain circumstances. In primary neuronal cultures from WT mice as well as cell lines overexpressing disease-related proteins, the PFFs can cause synaptic dysfunction, changes in cell excitability, and cell death.^206,207^ In mice overexpressing disease-related proteins or non-transgenic animals, intracerebral injection of PFFs into the dorsal striatum causes dysregulation of striatal DA release, neurodegeneration in the SNpc, and behavioral impairments.^208–211^ This type of model exhibits a longer time course of degeneration than other models, with early α-synuclein pathology in PD-relevant brain regions and the development of DA dysfunction, nigral degeneration, and motor deficits months after induction, suggesting a progression that is similar to that of the human condition. The repeatability of the results and the investment in a model with a time course of disease that takes several months to develop have been hampered by the model’s inability to reliably generate pathogenic PFFs.

## Therapeutic strategies for PD

### Commercially available drugs for PD

Many drugs have emerged as appropriate treatments, we differentiate the drugs according to their pharmacological targets and list them in Table 1.

**Table 1:** Commercially available drugs for PD treatment

Category	Drug	Therapeutic applications	Common side effects other than dyskinesia
L-DOPA preparation	L-DOPA/benserazide tablet	Parkinson’s syndrome	Exercise complications, nausea, vomiting, loss of appetite, postural hypotension, mental disorders, cardiac arrhythmias
	Carbidopa/L-DOPA tablet	Parkinson’s syndrome	
	Carbidopa/L-DOPA controlled-release tablet	Parkinson’s syndrome, wearing-off, Dyskinesia	Nausea, movement disorders, psychosis, dizziness, hallucinations, chorea, dystonia, drowsiness, insomnia, depression, vomiting, loss of appetite
DA agonists	Pramipexole tablet	Parkinson’s early syndrome, L-DOPA adjunct, wearing-off, Dyskinesia	Nausea, vomiting, constipation, hypotension, peripheral edema, vertigo, drowsiness, insomnia and hallucinations, confusion
	Ropinirole tablet	Parkinson’s early syndrome, L-DOPA adjunct, wearing-off, Dyskinesia	Nausea, vomiting, constipation, hypotension, peripheral edema, drowsiness, hallucinations, confusion, impulse control disorder
	Piribedil	Tremor, DA adjunct	Nausea, vomiting, constipation, dizziness, sleep disorders, hallucinations, impulse control disorders
	Transdermal rotigotine	Parkinson’s early syndrome, L-DOPA adjunct, wearing-off, Dyskinesia	Administration site reactions, nausea, vomiting, constipation, drowsiness, hypotension, peripheral edema, dizziness, bradykinesia, impulse control disorder
	Injected apomorphine	Wearing-off, L-DOPA-induced dyskinesias	Administration site reactions, dizziness, hypotension
MAO-B inhibitors	Selegiline	Parkinson’s early syndrome, wearing-off, Dyskinesia	Nausea, elevated liver enzymes, confusion, abnormal movement, bradycardia, L-DOPA side effects enhancement, insomnia, dizziness
	Rasagiline	Parkinson’s early syndrome, L-DOPA adjunct, wearing-off, Dyskinesia	Dyskinesia, nausea, dry mouth, vomiting, hallucinations, upright hypotension, musculoskeletal pain
	Safinamide	Wearing-off, Dyskinesia	Motor dysfunction, falls, nausea, insomnia, postural hypertension, anxiety, cough, indigestion
	Zonisamide	Wearing-off	Dizziness, irritability, depression, hallucinations, balance disorders, nausea, vomiting, stomach pain, diarrhea
COMT inhibitors	Entacapone	Wearing-off, Dyskinesia	Hypermobility, nausea, diarrhea, headache, abdominal pain, sleep disturbances, hallucinations
	Opicapone	Wearing-off, Dyskinesia	Drowsiness, hypotension, movement disorders, hallucinations, impulse control disorders, withdrawal reactions
	Tolcapone	Wearing-off	Dyskinesia, nausea, sleep disturbance, anorexia, drowsiness, confusion, dizziness, vomiting
Anticholinergics	Benztropine	Parkinson’s early syndrome, L-DOPA adjunct	Dizziness, memory loss, blurred consciousness, drowsiness, hallucinations, nausea
	Trihexyphenidyl	Parkinson’s early syndrome, L-DOPA adjunct	Dizziness, memory loss, blurred consciousness, drowsiness, hallucinations, nausea
Adenosine A2a receptor antagonists	Istradefylline	Wearing-off	Movement disorders, dizziness, constipation, nausea, hallucinations, insomnia
N‑methyl-d‑aspartate receptor antagonist	Amantadine	Parkinson’s early syndrome, L-DOPA adjunct	Depression, congestive heart failure, upright hypotension, psychosis, urinary retention, thrombocytopenia
Others	Clozapine	Dyskinesia	Cardiotoxicity, granulocyte deficiency, constipation, hypersalivation, drowsiness, dizziness, tremors, hyperkinesia, withdrawal reactions

### Levodopa

Although a classic treatment for PD,^212,213^ levodopa (L-DOPA) has several undesirable side effects, including motor-response oscillations and drug-induced dyskinesias. Both presynaptic and postsynaptic mechanisms are involved in the development of these motor complications, which eventually arise from non-physiological pulsatile striatal DA receptor stimulation.^214,215^ The key cause of maladaptive neuronal responses is discontinuous drug delivery, stemming from L-DOPA’s short half-life, as well as variability in gastrointestinal absorption and blood brain barrier transport. To address these challenges, novel sustained-release formulations of L-DOPA and continuous delivery techniques are continuously being developed. These include intestinal delivery via percutaneous endoscopic gastrojejunostomy tubes and subcutaneous delivery via minipumps.^216^

### DA agonists

Striatal medium spiny neurons have two types of DA receptors. Receptor agonists that directly target the D2 receptor family include dopaminomimetics such as the ergot alkaloid bromocriptine.^217,218^ Ergot alkaloids are ergoline derivatives that also activate 5-hydroxytryptamine (5-HT) receptors, including the 5-HT_2B_ subtype. However, they have been implicated in cardiac valvular fibrosis and pleuropulmonary fibrosis, raising important safety concerns. In contrast, non-ergoline drugs do not have this issue and are thus preferred for PD treatment. DA agonists have a longer half-life than L-DOPA, making them strong candidates for adjunct therapy in patients with motor fluctuations.^218–220^ However, they have a lower overall effect than L-DOPA, as well as a higher tendency to cause sleepiness and hamper impulse control.^221^ Apomorphine is unique among DA agonists in having combined action on both D1 and D2 receptors, along with an equal affinity for L-DOPA.^222^ Continuous subcutaneous apomorphine infusions have been linked to a decrease in pre-existing L-DOPA-induced dyskinesias and limiting motor-response variations.^223^ Currently, new apomorphine formulations for sublingual use are undergoing clinical development.^224^

### Catechol-O-methyltransferase and monoamine oxidase type B inhibitors

During the peripheral metabolism of L-DOPA, catechol-O-methyltransferase (COMT) ortho-methylates the drug via a secondary metabolic route. When COMT is inhibited, L-DOPA bioavailability and half-life are improved.^225^ Given this effect, COMT inhibitors combined with L-DOPA have become part of the first-line treatment for patients with PD. Currently, three COMT inhibitor preparations are available for clinical use, including entacapone and opicapone.^217,226^ Monoamine oxidase type B (MAO-B) is a primary clearance mechanism for synaptically released DA in glial cells.^227^ Inhibiting MAO-B (e.g., through selective inhibitor selegiline) prolongs DA’s effect and increases its synaptic concentrations.^228^ However, because selegiline is irreversible, safinamide has emerged as a reversible MAO-B inhibitor for use in PD treatments.^229^

### Non-dopaminergic targets

Despite the remarkable effect of dopaminergic therapy on PD symptoms, therapies involving other targets remain a necessity. First, novel treatments are required to address complications of L-DOPA therapy, such as motor fluctuations, L-DOPA-induced dyskinesia, and L-DOPA-resistant (“non-dopaminergic”) motor features (e.g., treatment-resistant tremors, postural instability, frozen gait, swallowing difficulties, and speech disturbances). Currently, the only accessible and effective pharmacological tool for L-DOPA-induced dyskinesia is amantadine, hypothesized to be a N-methyl-D-aspartate receptor antagonist.^217,220^ Second, novel treatments must also address non-motor symptoms of PD, particularly depression, cognitive dysfunction, and autonomic failure. A major problem with non-motor symptoms is that many are unresponsive to DA replacement therapy; some are even precipitated or aggravated by this treatment.^230^ Cholinesterase inhibitors have beneficial effects on cognitive disturbances in patients with PD and dementia. This positive outcome is possibly associated with a significant loss of cholinergic projections from the nucleus basalis of Meynert in dementia.^217,231^ For psychotic symptoms in PD, clozapine is the most effective therapy. Finally, autonomic dysfunction is quite common in PD, especially during the late stage. A number of pharmacological therapies are available that predominantly focus on the autonomic nervous system. These include mineralocorticoid fludro cortisone; adrenergic agents (e.g., midodrine and etilefrine); anti-muscarinics (e.g., tolterodine, oxybutynin, or trospium chloride) for urinary urgency or incontinence; noradrenaline precursor (droxidopa) to treat orthostatic hypotension; and prokinetic drugs (e.g., macrogol or lubiprostone) to improve constipation.^218,219,232^

## Drugs for PD treatment under clinical trials

Numerous clinical trials are underway to test the novel drugs that have been developed (Table 2). Some of them have shown potential candidates for PD. Tavapadon is a potent, highly selective, orally administered, DA D1/D5 receptor partial agonist and in the clinical trial (NCT02847650), compared to placebo, Tavapadon showed a better improvement effect which provided the research basis for the current clinical phase III trial (NCT04760769).^233^ IRL790 could interact with DA D3 receptor, and it was developed as an experimental treatment for L-DOPA-induced dyskinesia, impulse control disorder, and psychosis in PD. In the research,^234^ patients with advanced PD on IRL790 experienced a reduction in motor symptoms and no serious adverse effects and in the follow-up phase II clinical trials are still in the early stages (NCT03368170). Deferiprone in Phase II randomized double-blind placebo-controlled clinical trials (NCT00943748, NCT01539837) reduced SN iron deposition and progression of motor handicap in PD patients.^235,236^ Cu(II)ATSM exerted a positive effect by preventing lipid peroxidation in a Phase I dose-escalation study in early PD patients (NCT03204929) which suggests the potential for PD treatment.^237^ Prasinezumab is a monoclonal antibody directed against α-synuclein. As a potential therapeutic approach against a key target in PD, its development has received much attention but in a new phase II clinical study (NCT03100149), it showed no therapeutic effect compared to placebo and has safety concerns.^238^ But its effects may require more experiments to verify, and new clinical trials are underway (NCT04777331). DNL151 is an LRRK2 inhibitor and has shown a relatively obvious therapeutic effect on PD in the double-blind randomized clinical phase I trial (NCT04056689) hosted by Denali Therapeutics, and now it has launched clinical phase II (NCT05348785) and III phase trials (NCT05418673). If the results are good, it may prove the great potential of LRRK2 in PD treatment.

**Table 2:** Clinical therapeutic interventions for PD treatment

Therapeutic strategy	Name	Classification and target	Status	ClinicalTrials.gov Identifier
DA receptor agonists	PF-06649751/CVL 751/ Tavapadon	Small molecular DA D1/D5 agonist	Phase III	NCT04223193, NCT04542499, NCT04201093
	PF-06669571	Small molecular DA D1 agonist	Phase I	NCT02565628
	PF-06412562	Small molecular DA D1 agonist	Phase I	NCT03665454
	KDT-3594/AM-006	Small molecular DA agonist	Phase II	NCT04867551, NCT03845387
	Lu-AF28996	Small molecular DA D1/D2 agonist	Phase I	NCT04291859
Anti-α-synuclein aggregation therapy	Prasinezumab/PRX002/RO7046015	Monoclonal antibody	Phase IIB	NCT04777331
	MEDI-1341/TAK-341	Monoclonal antibody	Phase I	NCT04449484
	Lu AF82422	Monoclonal antibody	Phase II	NCT05104476
	UCB7853	Monoclonal antibody	Phase I	NCT04651153
	UCB 0599	Small molecular SNCA antagonists	Phase II	NCT04658186, NCT05543252
	Kenterin/Enterin-01	Small molecular SNCA antagonist	Phase II	NCT04483479
	Ambroxol	Small molecular Decrease the cerebrospinal fluid α-synuclein level	Phase II	NCT02914366
Targeting ferroptosis	Cu(II)ATSM	Small molecular Peroxynitrite scavenger	Phase I	NCT03204929
	DFP/Deferiprone	Small molecular	Phase II	NCT01539837
Serotonin receptor agonists or antagonists	Landipirdine/SYN120/RO-5025181	Small molecular Dual 5-HT6/5-HT2 antagonist	Phase II	NCT02258152
	SEP-363856	Small molecular 5-HT1A agonist	Phase II	NCT02969369
N-methyl-d-aspartate receptor (NMDAR) modulators	NBTX 001	Small molecular NMDAR modulator	Phase I	NCT04097080
	NYX-458	Small molecular NMDAR modulator	Phase II	NCT04148391
	DAAOI-P	Small molecular D-amino acid oxidase inhibitor	Phase II	NCT04470037
Adenosine A2a receptor antagonists	KW-6356	Small molecular Adenosine A2A receptor antagonist	Phase IIB	NCT03703570
	Caffeine	Small molecular Selective Adenosine A2A antagonist	Phase III	NCT01738178
Acetylcholinesterase antagonists	Buntanetap/ANVS-401	Small molecular AchE antagonist/TAU antagonist	Phase III	NCT05357989
Muscarinic and nicotinic acetylcholine receptor agonists	Blarcamesine/AF710B/ANAVEX 2-73	Small molecular Muscarinic acetylcholine receptor M1 agonist	Phase II	NCT04575259
	Nicotine nasal spray	Small molecular Nicotinic agonist	Phase II	NCT03865121
Kinase inhibitors	SUN-K706/Vodobatinib/SCC-138/K0706	Small molecular Bcr-Abl antagonist	Phase II	NCT03655236
	Nilotinib/Tasigna/AMN-107	Small molecular Bcr-Abl antagonist	Phase II	NCT03205488
	Radotinib Dihydrochloride/ IY-5511	Small molecular Bcr-Abl antagonist	Phase II	NCT04691661
	BIIB-122/DNL151	Small molecular LRRK2 antagonist	Phase III	NCT05418673
	DNL-201	Small molecular LRRK2 antagonist	Phase I	NCT03710707
Botanical-based medication	DA 9805	Natural compounds	Phase II	NCT03189563
	Hypoestoxide	Plant-based herbal dry powder	Phase I/ II	NCT04858074
	WIN-1001X	Plant-based herbal extract	Phase II	NCT04220762
Cell-based therapy	NTCELL	Immunoprotected (alginate-encapsulated) porcine choroid pplexus cells	Phase I/ II	NCT01734733
	ISC-hpNSC	Neural stem cells	Phase I	NCT02452723
	ANGE-S003	Neural stem cell	Phase II/ III	NCT03128450
Gene therapy	AAV2-GDNF	AAV2-GDNF delivered to the putamen	Phase I	NCT04167540
	LY3884961/PR001A	Glucocerebrosidase gene therapy by intra cisterna magna administration	Phase I/ II	NCT04127578
Others	CNM-Au8	Small molecular	Phase II	NCT03815916
	NLY01/NLY01-AD	Small molecular GLP1R agonist	Phase II	NCT04154072

## Botanical medications for PD treatment

**Fig. 5:**
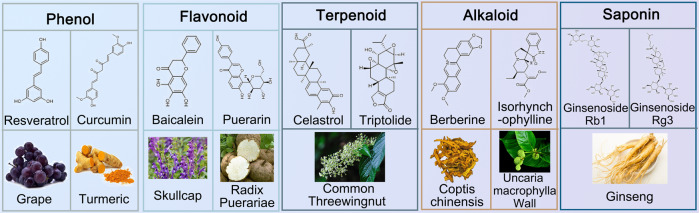
Natural compounds for PD treatment derived from traditional Chinese medicine

Given the side effects of Western medicine and the invasiveness of external physical interventions, new treatments should be developed. In the past few years, many researchers have investigated the role of various natural products from medicinal plants and their formulations in the treatment of PD. Numerous natural products have been identified for the molecular regulation of PD (Table 3 and Fig. 5).

**Table 3:** Natural compounds with therapeutic potential for PD treatment

Category	Name	Formula	Pharmacological effects	Major targets
Phenol	Curcumin	C_21_H_20_O_6_	TNF-α ↓ , caspase activity ↓ , inflammatory cytokines ↓ , inflammatory enzymes ↓ , cycloxygenase-2 ↓ , GFAP ↓ , cyclin D1 ↓ , JNK phosphorylation ↓ , cell apophagy ↓ , BDNF ↑	Trk/PI3K, JNK
	Resveratrol	C_14_H_12_O_3_	Cytochrome C ↓ , apoptosis ↓ , ROS ↓ , α-synuclein ↓ , autophagic flux ↑ , Bax/Bcl-2 ↑ , antioxidant defenses↑	PI3K/Akt, SIRT 1, MAPK
Alkaloid	Berberine	C_20_H_18_NO_4_^+^	Neurons degeneration ↓ , motor impairment ↓ , α-synuclein ↓ , autophagy ↑ , L-DOPA ↑	AMPK,
	Isorhynchophylline	C_22_H_28_N_2_O_4_	α-synuclein ↓ , autophagy↑	ASK1/JNK
Flavonoid	Puerarin	C_21_H_20_O_9_	Caspase-3 ↓ , p53↓	Fyn/GSK-3β, PI3K/Akt
	Baicalein	C_15_H_10_O_5_	Caspase-3 ↓ , α-synuclein ↓ , neuroinflammation ↓ , ROS ↓ , Bax/Bcl-2↑	NLRP3/caspase-1/gasdermin D, BDNF/TrkB/CREB
Terpenoid	Celastrol	C_29_H_38_O_4_	Neuroinflammation ↓ , motor symptoms ↓ , neurodegeneration ↓ , autophagy ↑ , autophagosome biogenesis↑	MAPK, Nrf2-NLRP3-caspase-1
	Triptolide	C_20_H_24_O_6_	Microglial activation ↓ , proinflammatory cytokines ↓ , α-synuclein↓	miR155-5p/SHIP1, NF-κB
Saponin	Ginsenoside Rb1	C_54_H_92_O_23_	hippocampal CA3 α‐synuclein ↑ , glutamate ↑ , DA ↑ , neuroinflammation↓	NF-κB
	Ginsenoside Rg3	C_42_H_72_O_13_	DA ↑ , ROS ↓ , hydroxylase-positive neurons↑

### Phenol

Using a 6-OHDA-induced PD model, Yang et al. demonstrated the protective effects of curcumin on the injured hippocampus. These benefits included significant improvement in mental status, increases to DA and norepinephrine levels, neural regeneration of hippocampal tissue, and activation of proteins involved in cell survival-related pathways, such as BDNF, tropomyosin receptor kinase (Trk) B, and phosphoinositide 3-kinase (PI3K).^239^ Corroborating this study, other experiments with 6-OHDA PD models have shown that curcumin restores neuronal regeneration via stimulating Trk/PI3K signaling, which limits TNF-α and caspase activity while increasing BDNF levels.^240^ Further research into curcumin’s mechanism of action indicates that it at least partially involves interacting with a α7 nicotinic acetylcholine receptor. Through this mechanism, curcumin enhances the survival of striatal TH fibers and neurons in the SNpc and decreases abnormal turning behavior.^241^ Curcumin also inhibits a wide range of inflammatory compounds, including cytokines, ILs, chemokines, inflammatory enzymes, cycloxygenase-2, glial fibrillary acidic protein, and cyclin D1. Furthermore, curcumin blocks numerous participants in apoptotic pathways, such as iNOS, LPS-induced TNF-α, IL-1β, and IL-6, and c-Jun N-terminal kinase (JNK).^242–245^ These anti-inflammatory properties are further verified with data showing that curcumin modulates of the effects of various inflammatory mediators.^246,247^ In vitro and in vivo studies have shown that resveratrol exerts neuroprotective effects in PD models generated from 6-OHDA, MPP^+^, and rotenone.^248–251^ Resveratrol activates the pro-survival PI3K/protein kinase B (Akt) pathway, increases the B-cell lymphoma (Bcl-2)/Bcl-2-associated X (Bax) ratio, and decreases cytochrome C release so that caspase-3 remains inactive, thereby blocking apoptosis.^252^ Furthermore, resveratrol decreases ROS production and increases antioxidant defenses after exposure to MPTP/MPP^+^^253^ and rotenone.^254,255^ In experimentally induced PD, resveratrol protects against mitochondrial dysfunction, counteracting changes to mitochondrial morphology and mitochondrial membrane potential,^250,254^ while increasing mitochondrial biogenesis and complex-I activity.^255,256^ In several animal models, resveratrol stimulates autophagic degradation of α-synuclein after sirtuin (SIRT) 1 activation and decreases α-synuclein expression in the striatum.^257,258^ Another neuroprotective mechanism of resveratrol is similar to gastrodin action, activating HO-1 and mitogen-activated protein kinase (MAPK) pathways to increase autophagic flux.^254,259^ Regulation of astroglial activation also plays a role in neuroprotection. In a promising sign for clinical application, resveratrol presents synergistic effects when administered with L-DOPA.^258^

### Alkaloids

Experiments applying berberine on MPTP-treated mice significantly ameliorated dopaminergic neuronal degeneration in the SN compacta and improved motor impairment. Berberine also decreased α-synuclein levels, enhanced microtubule-associated protein light chain 3 (LC3-II)-associated autophagy. Furthermore, berberine also activated adenosine 5′-monophosphate (AMP)-activated protein kinase (AMPK), and a notable benefit is that AMPK lowers α-synuclein-induced toxicity and protects cells from rotenone.^260–262^ Another study investigating the effects of berberine in mouse models showed that NLRP3-associated neuroinflammation was significantly mitigated and decreased the level of NLRP3 inflammasome in mice treated with berberine.^263^ The specific mechanism of berberine action may be its effect on TH, the rate-limiting enzyme in the phenylalanine–tyrosine–DA pathway. This pathway provides DA to the brain and generates L-DOPA with tetrahydrobiopterin as a coenzyme. In the gut, bacterial nitroreductase has been shown to transform berberine into dihydroberberine. This reaction supplies H• and increases tetrahydrobiopterin concentrations, which in turn enhances TH activity. The end result is an acceleration of L-DOPA production by gut bacteria.^144,264,265^ Through the autophagy–lysosome pathway, isorhynchophylline (IRN) promotes the clearance of WT, A53T, and A30P α-synuclein aggregates in neuronal cells. While independent of the mTOR pathway, IRN-induced autophagy is dependent on Beclin 1 function.^266^ Treatment with IRN markedly reduced MPP^+^-induced endoplasmic-reticulum stress responses. In addition, IRN inhibition of the apoptosis signal-regulating kinase 1 (ASK1)/JNK pathway appears to suppress mitochondria-dependent apoptosis which suggests the protection of neurons.^267–270^

### Flavonoids

The flavonoid puerarin has anti-parkinsonian effects that are dependent on Nrf2. In a study using MPTP-treated mice, puerarin regulated Fyn and GSK-3β phosphorylation in the ventral midbrain. The Fyn/GSK-3β pathway facilitates Nrf2 accumulation in the nucleus, leading to de novo glutathione synthesis.^271^ Available evidence indicates that puerarin promotes dopaminergic neuron survival, proliferation, and differentiation via progesterone receptors.^272,273^ Puerarin also acts on the PI3K/Akt pathway to alleviate inflammatory responses^274^ and inhibit GSK-3 activity in neurons, thus limiting caspase-3 production and associated apoptosis.^275,276^ These interactions, along with inhibition of nuclear p53 accumulation, explain how puerarin protects against MPP^+^-induced neuroblastoma SH-SY5Y cell death.^277^ Baicalein administration reversed MPTP-induced motor dysfunction, dopaminergic neuronal loss, and proinflammatory cytokine elevation. In addition, baicalein inhibited the activation and proliferation of disease-associated proinflammatory microglia. The underlying mechanism of this protective effect is probably inhibition of the NLRP3/caspase-1/gasdermin D pathway.^278^ This pathway is associated with pyroptosis, a type of programmed cell death that participates in the loss of dopaminergic neurons.^279–282^ Pytoptosis occurs first through activating the NLRP3 inflammasome, which then promotes caspase-1 maturation.^283^ Caspase-1 then mediates the oligomerization of gasdermin D, a pyroptosis executive protein, thus stimulating proinflammatory IL-1β and IL-18 secretion.^284,285^ Research also showed that baicalein acts on the BDNF/TrkB/Cyclic AMP response-element binding protein (CREB) pathway to reduce α-synuclein aggregation and protect synaptic plasticity.^286^ Several studies using baicalein under different conditions have further highlighted its therapeutic value. For instance, combining baicalein and low-dose L-DOPA significantly recovered gait function in patients to a level comparable with results from high-dose L-DOPA treatment, although some L-DOPA side effects were also present.^287^ Finally, baicalein antagonized rotenone-induced ROS overproduction, upregulated Bax and cleaved caspase-3, downregulated Bcl-2, and phosphorylated extracellular signal-regulated kinases (ERK) 1/2.^288^

### Terpenoids

Through acting on the Nrf2-NLRP3-caspase-1 pathway to inhibit the NLRP3 inflammasome, celastrol relieves motor deficits and nigrostriatal dopaminergic degeneration.^289^ In neurons, celastrol promotes autophagy, autophagosome biogenesis, and mitophagy, probably in association with MAPK pathways. In MPP^+^-induced PD cell models, celastrol inhibits dopaminergic neuronal death, ATP loss, and mitochondrial membrane depolarization. Research using these models also suggest that celastrol maintains mitochondrial quality by sequestering defective mitochondria into autophagosomes for degradation.^290^ After α-synuclein preformed fibril-induced microglial activation, triptolide treatment suppressed microglial activation and attenuated proinflammatory cytokine release. Specifically, the drug targeted the miR155-5p/Src homology 2 (SH2) domain in NF-*κ*B, suppressing its activity in the inositol polyphosphate 5-phosphatase (SHIP)1 pathway.^291^ Studies suggest that miR155-5p overexpression provokes NF-*κ*B activity through SHIP1 suppression.^292^ Triptolide acts to disrupt miR155-5p repression of SHIP1, thereby mitigating the inflammatory reaction. Research in an LPS-induced PD model demonstrated that blocking metabotropic glutamate receptor subtype 5 (mGlu5) attenuated the anti-inflammatory effects of triptolide. In addition, mGlu5 appears to mediate the effect of triptolide on microglia-induced astrocyte activation in vitro and in vivo.^293^ Triptolide has also been described as a potent autophagy inducer in neuronal cells, helping to clear various forms of α-synuclein via the autophagic pathway.^294^

### Saponins

The underlying neuroprotective mechanism of ginsenoside Rb1-improvements to synaptic plasticity involves promoting hippocampal CA3 α-synuclein expression, restoring glutamate in the CA3‐schaffer collateral-CA1 pathway, and sequentially increasing postsynaptic density-95 expression.^295^ In LPS-treated rats, ginsenoside Rb1 treatment considerably lowered apomorphine-induced rotations, SN inflammation, and DA (plus metabolites) depletion in the striatum. These effects may be related to the inhibition of the NF-*κ*B signaling pathway.^296^ The ginsenoside Rg3 augmented TH-positive neuron count in the SN, mean density of TH-positive nerve fibers, and DA content in the striatum, while also lowering ROS levels in the SN.^297^

## Discussion and perspectives

PD is a debilitating neurodegenerative disease with pathological hallmarks of α-synuclein accumulation and loss of dopaminergic neurons in SN. The mechanisms involved in PD are very complex, such as the aggregation of α-synuclein, OS, neuroinflammation, ferroptosis, mitochondrial dysfunction, gut dysbiosis, etc. Interactions also have an important impact on the occurrence and progression of PD. The deep mechanism of the impact of these mechanisms on PD still needs to be explored, and for the models needed for experimental research, in addition to the toxin model, transgenic model and PFFs model that we have summarized, new models are needed to promote the research of PD. In the strategy for the treatment of PD, the main drug is still L-DOPA, and other drugs such as MAO-B inhibitors, COMT inhibitors, etc. are more commonly used in combination with L-DOPA. Moreover, the existing drugs often have no effective therapeutic effect on patients with advanced PD, which greatly reduces the quality of life of PD patients. Therefore, new drugs are still needed for treatment. Exploring new drugs can be considered from the mechanism of PD, such as targeting α-synuclein aggregation, ferroptosis and OS etc. The related drugs currently under development have not shown good effects, and may still require a long period of exploration. As for the new PD treatment drugs, besides the current hot chemical drugs, biological drugs, etc., some researchers have approved the therapeutic efficacy of traditional Chinese medicines which show tremendous potential in the field of PD treatment. The compounds derived from traditional Chinese medicines have complicated pharmacological effects and reverse the pathological mechanisms of PD such as the OS, neuroinflammation, and aggregation of α-synuclein. In the review of Sun et al., numerous natural drugs such as tanshinone and andrographolide that are reported for the anti-inflammatory effects and may be potential drugs for the treatment of PD have been proposed for further investigations.^265^ Moreover, based on the complex mechanisms, multi-drug combinations may offer a new perspective on PD treatment such as the combination of biological and chemical drugs or natural small molecules.

Besides the drug interventions, some surgical treatment methods also carry patients' new optimal approach. Deep brain interference induced by high-frequency (100–200 Hz) electrodes can replicate the impact of a lesion without impairing brain tissue.^219^ Taking advantage of this technology, a clinical trial has combined transcranial direct current stimulation and treadmill gait training to improve gait-function recovery in PD patients (NCT04591236). Transcutaneous magnetic stimulation of the spinal cord has also emerged recently as a possible therapeutic option for gait disorders, capable of stimulating neural elements non-invasively (NCT05008289). Another promising area of research is the use of stem cells. A clinical study first differentiated patient-derived midbrain dopaminergic progenitor cells from autologous iPSCs. These cells were then implanted into the putamen. The PD symptoms of treated patients then improved, allowing for a 6% decrease in L-DOPA-equivalent daily dose.^298^ These suggest that researchers may discovery the novel treatment approach in the area of stem cell transplant.
